# Hepatitis B and D burden in Uzbekistan: comprehensive analysis of seroprevalence and risk correlates from a countrywide study

**DOI:** 10.1186/s40249-026-01487-w

**Published:** 2026-08-17

**Authors:** Erkin Isakovich Musabaev, Malika Erkinovna Khodjaeva, Aziza Saydullaevna Khikmatullaeva, Nargiz Sapievna Ibadullaeva, Fatima Shokir kizi Shodieva, Gulnara Karimovna Khudaykulova, Nigora Ubaydullaevna Tadjieva, Sobirjon Bakhtiyorovich Amonov, Ulugbek Khudayberdievich Mirzaev

**Affiliations:** 1 Department of Hepatology, Research Institute of Virology, Tashkent, Uzbekistan; 2 Department of Public Health and Healthcare Management, Tashkent State Medical University, Tashkent, Uzbekistan; 3 Department Biomedical Sciences, Pharmaceutical Technical University, Tashkent, Uzbekistan; 4 Department of Epidemiology, Tashkent State Medical University, Tashkent, Uzbekistan; 5 Department of Infectious Disease, Dermatology, Phthisiatry and Pulmonology, Termez Branch of Tashkent State Medical University, Termez, Uzbekistan; 6 Department of Emerging Infectious Diseases, Research Institute of Virology, Tashkent, Uzbekistan; 7 Department of Microbiology and Antimicrobial resistance, National Biopharmaceutical Research Institute, 1, Bog’zor, 111800 Tashkent, Uzbekistan

**Keywords:** Hepatitis B, Hepatitis D, Risk factor, Serological test, Uzbekistan

## Abstract

**Background:**

Uzbekistan faces a substantial burden of hepatitis B and D virus (HBV and HDV) infections despite implementing universal hepatitis B vaccination in 1998. Regional variation in disease distribution and contemporary transmission dynamics remain inadequately characterized. Our objective was to evaluate the geographically stratified prevalence of HBV and HDV, while also identifying the key risk factors fueling transmission within the adult population of Uzbekistan.

**Methods:**

We conducted a nationwide cross-sectional study of 10,552 adults across seven regions of Uzbekistan from July 2022 to June 2024. Participants were selected using stratified random sampling from a parent cohort of 1,048,575 individuals. Serological testing included hepatitis B surface antigen (HBsAg), antibodies to hepatitis B core antigen (anti-HBc), anti-HDV antibodies, and viral RNA detection. Multivariate logistic regression identified independent risk factors for HBV and HDV infections.

**Results:**

Overall HBsAg prevalence was 4.7% (95% *CI*: 4.3–5.1%), with substantial geographic heterogeneity ranging from 3.2% in Tashkent to 6.3% in Bukhara. Among HBsAg-positive individuals, 68.1% had detectable HBV DNA and 12.7% had HDV superinfection. HDV RNA prevalence among HBV DNA-positive individuals ranged from 2.5% to 23.7% across regions. Age-cohort analysis demonstrated vaccination program impact, with older unvaccinated adults bearing disproportionate disease burden. Female sex associated with 31% lower odds of chronic HBsAg carriage. Married status, healthcare-associated exposures, and body piercings correlated with increased anti-HBc positivity. Remarkably, sharing personal hygiene items showed striking HDV-specific association (a*OR* 2.34, 95% *CI*: 1.16–4.74; *P* = 0.02) but no association with HBV markers, suggesting distinct transmission pathways for HDV superinfection.

**Conclusions:**

Persistent HBV burden with notable regional clustering and HDV superinfection necessitates targeted interventions. The identification of personal hygiene item sharing as a specific HDV transmission route provides an actionable household-level prevention target. Intensified screening and treatment of unvaccinated adults remain critical for hepatitis elimination efforts.

**Supplementary Information:**

The online version contains supplementary material available at 10.1186/s40249-026-01487-w.

## Background

Hepatitis B virus (HBV) remains a significant global public health challenge, with an estimated 250 million chronically infected individuals worldwide who face substantial risks of cirrhosis, hepatocellular carcinoma (HCC), and liver-related mortality [1]. The burden of chronic HBV infection is heterogeneously distributed, with the highest prevalence concentrated in regions of Asia, Africa, and parts of Eastern Europe. Central Asia, including Uzbekistan, historically has experienced intermediate to high endemicity of HBV infection, though the epidemiological landscape has been substantially transformed by the introduction of universal hepatitis B vaccination programs over the past three decades [2–5].​

Hepatitis D virus (HDV) infection represents a critical complication of chronic HBV infection, occurring only in individuals with evidence of hepatitis B exposure or active viremia. Globally, approximately 4.5% of hepatitis B surface antigen (HBsAg)-positive individuals are estimated to carry HDV infection, affecting nearly 12 million people worldwide and representing a major contributor to HBV-associated liver disease complications. HDV superinfection of persons with chronic HBV infection is considered the most severe form of chronic viral hepatitis due to accelerated progression to cirrhosis and heightened risk of hepatocellular carcinoma compared to HBV infection alone, with HDV infection accounting for approximately 18–20% of cirrhosis and HCC cases among HBsAg-positive populations. The geographic distribution of HDV remains profoundly heterogeneous, with particularly high prevalence documented in Mongolia, the Republic of Moldova, and countries in Western and Central Africa, suggesting region-specific transmission dynamics that remain incompletely characterized [6–8].

Uzbekistan, situated at the intersection of major Central Asian trade and migration routes, has faced historically significant burdens of both HBV and HDV infection. The country’s national HBV vaccination program, launched in 1998 and achieving full population coverage by 2000, represents one of the region’s most comprehensive public health interventions targeting infectious disease prevention. Previous surveillance data documented that HBsAg prevalence declined dramatically from 3.2% in individuals born before 2000 to 0.8% in post-2000 birth cohorts, providing compelling evidence for the vaccination program's effectiveness in preventing infection in younger generations. However, substantial challenges persist and the country continues to face endemic HDV circulation [2, 3, 9].​

Despite Uzbekistan's noteworthy progress in HBV prevention through vaccination, several critical knowledge gaps remain. First, the geographic distribution of both HBV and HDV infection across Uzbekistan’s diverse administrative regions has not been comprehensively characterized in recent years. Regional variations in prevalence may reflect differences in vaccination coverage, healthcare infrastructure, infection control practices, or behavioral and demographic risk factors that could inform targeted public health interventions [8, 10]. Second, the epidemiological relationships between HBV and HDV infections remain inadequately understood in Central Asian populations. Most global HDV research has focused on high-risk populations (people who inject drugs, men who have sex with men, haemodialysis recipients) or specific geographic hotspots in Africa or East Asia, with limited investigation of HDV transmission pathways in the general adult populations of Central Asian countries [11]. Third, while previous studies have identified age-related cohort effects and documented the vaccination program's impact, the specific risk factors driving persistent HBV and HDV transmission in adults—particularly regarding household and healthcare-associated exposures—require contemporary investigation to guide prevention strategies.

To address these evidence gaps, we conducted a large population-based study of HDV testing and epidemiological risk factor analysis among 10,552 adults sampled from a comprehensive nationwide hepatitis B and C screening survey conducted across all regions of Uzbekistan from July 2022 to June 2024. By employing stratified random sampling from an exceptionally large parent cohort (*n* = 1,048,575 individuals screened at healthcare facilities), we aimed to provide representative prevalence estimates of HBV and HDV infections stratified by geographic region, and to identify specific risk factors driving HBV and HDV transmission in contemporary Uzbek adults [9]. Our findings provide critical epidemiological evidence to inform Uzbekistan's ongoing efforts toward viral hepatitis elimination, align with World Health Organization targets for HBV and HDV surveillance, and identify actionable household-level and healthcare-based prevention strategies applicable to Central Asian settings.

## Methods

### Study design and parent cohort

This study is part of a comprehensive nationwide hepatitis B and C screening survey conducted in Uzbekistan from July 2022 to June 2024, which has been previously described in detail [12]. Briefly, the parent study screened 1,048,575 individuals aged 1–95 years at local healthcare facilities across all 14 administrative regions of Uzbekistan using convenience sampling. Participants were individuals who visited local healthcare facilities, had no previous history of HBV or hepatitis C virus (HCV) infection, and voluntarily consented to participate in the screening program.

### Subsample selection

The subsample of 10,552 participants was selected from the parent cohort (*n* = 1,048,575) using stratified random sampling by administrative region. While the original cohort was recruited through convenience sampling at healthcare facilities during the nationwide screening campaign, the subsample selection employed probability-based methods to ensure representativeness. Specifically, we stratified the cohort by seven administrative regions where hepatitis D virus (HDV) testing was prioritized: Tashkent city, Tashkent region, Samarkand, Fergana, Andijan, Bukhara, and the Republic of Karakalpakstan. Within each regional stratum, participants were randomly selected using computer-generated random numbers (R software, version 4.3.2, R Core Team, Vienna, Austria), with allocation approximately proportional to regional population size. This resulted in sampling rates of 42–56 participants per 100,000 population across all regions, ensuring geographic representativeness of the subsample while maintaining adequate power for regional analyses. The final subsample comprised 1414 participants from Tashkent city, 1576 from Tashkent region, 1691 from Samarkand, 1986 from Fergana, 1811 from Andijan, 1011 from Bukhara, and 1060 from the Republic of Karakalpakstan.

### Sample size

Sample size calculations indicated that approximately 10,500 participants would provide 90% power to detect a 2% difference in HBsAg prevalence between regions (α = 0.05), assuming regional HBsAg prevalence rates of 3–6% based on preliminary screening data from the parent study. To account for potential missing data or incomplete serological records, we initially selected 11,000 participants, from which 10,552 had complete HBsAg, anti-HBc, and demographic data available for HDV antibody testing and subsequent analysis.

### Study population

The population of interest included individuals aged eighteen years and older who were registered in the parent cohort database. Inclusion criteria were: (1) age ≥ 18 years, (2) residence in the service areas of selected family clinics in the seven target regions, (3) availability of complete serological data (HBsAg and anti-HBc test results), and (4) adequate stored serum samples for HDV testing. Exclusion criteria included individuals with incomplete demographic or serological data, insufficient serum volume for additional testing, or improper sample storage conditions.

### Laboratory testing

All participants in the parent cohort had been initially tested for HBsAg using rapid immunochromatographic tests (InTecProducts, INC, Xiamen, China) at the point of care [13]. For the current subsample, stored serum samples were retrieved from − 20 °C storage at the Research Institute of Virology laboratory in Tashkent, Uzbekistan.

### Testing for HBV

All subsample serum specimens were retested for total antibodies to hepatitis B core antigen (anti-HBc) using the D-0566 VectoHBcAg test kit (Vector-Best, Novosibirsk, Russia) [14]. Samples testing positive for anti-HBc underwent confirmatory testing for HBsAg using the WHO-prequalified D-0544 HBsAg-EIA-BEST kit (Vector-Best, Novosibirsk, Russia) [14]. HBsAg-positive samples were subsequently tested for hepatitis B virus deoxyribonucleic acid (HBV DNA) using the Amplisens HBV-FRT PCR kit (Central Research Institute for Epidemiology, Moscow, Russia) [15], R-V5-Mod (RG, iQ, Mx, Dt)-CE or an in-house PCR system.

### Testing for HDV

All HBsAg-positive samples identified in the subsample were tested for total antibodies to hepatitis delta virus (anti-HDV) using the verified D-0954 Vectohep D antibody determination kit (Vector-Best, Novosibirsk, Russia), with preliminary verification performed by the reference laboratory group [16]. Samples testing positive for anti-HDV antibodies underwent hepatitis D virus ribonucleic acid (HDV RNA) testing using the Amplisens HDV-FL PCR kit, R-V3 (RG, iQ, Mx, Dt) (Central Research Institute for Epidemiology, Moscow, Russia) [17].

All laboratory procedures were conducted according to the manufacturers' instructions, and quality control measures were implemented throughout the testing process.

### Data collection

Demographic, clinical, and epidemiological data for the subsample were obtained from the parent study database [12]. In the parent study, data were collected by trained interviewers using structured questionnaires administered in Uzbek, Karakalpak, and Russian, and responses were entered electronically using tablet-based data collection forms.

Variables extracted for the present analysis included age, sex, region of residence, and selected exposure variables. History of invasive medical procedures was defined as any self-reported lifetime history (ever vs never) of surgical operations, endoscopic procedures, blood transfusions, dental procedures involving instruments, or therapeutic/diagnostic injections administered in a clinical setting. Sharing personal hygiene items was defined as self-reported regular within-household sharing of items that may meet blood or mucous membranes, including razors, toothbrushes, nail scissors or clippers, or similar personal grooming instruments. These variables were recorded as binary yes/no responses in the structured questionnaire.

Quality control procedures for the questionnaire survey were implemented at multiple levels. The structured questionnaire was adapted from the nationwide hepatitis B and C screening program and reviewed by hepatology, epidemiology, and public health experts to ensure content validity. Interviewers (nurses, physicians, and trained researchers) received standardized training on questionnaire administration, tablet-based data entry, and handling of sensitive questions before fieldwork. Supervisors performed on-site checks of a random subset of interviews and monitored data completeness and consistency in real time via the electronic system.

### Statistical analysis

JMP software version 18.0.1. (SAS Institute, Cary, NC, USA) was employed for all data analyses. Descriptive statistics were calculated for demographic characteristics and seroprevalence rates. Proportional estimates were calculated using Wald's 95% confidence intervals (*CI*). To determine independent risk factors for hepatitis B and delta virus infections among adults, we applied chi-square (*χ*^2^) test and multivariate logistic regression analysis. The analysis incorporated a stepwise selection approach, considering 11 questionnaire variables, including sex, age, level of education, marital status, occupation type, household size, history of surgical interventions, tattoos, piercings, use of intravenous drugs, and habits of sharing personal hygiene items within the family. A two-stage significance threshold approach was employed: a liberal screening criterion of *P* < 0.25 was used to identify candidate variables for multivariable analysis, while a conventional significance level of *P* < 0.05 was applied for determining final statistical significance in all other tests. The use of *P* < 0.25 as a univariate screening threshold follows the purposeful variable selection approach, which recommends a more liberal cutoff at the screening stage to avoid excluding potentially important confounders that may not show strong univariate associations but may contribute meaningfully to multivariable analysis [18, 19].

## Results

The study included 10,552 adults from seven regions of Uzbekistan, with 62.1% females and 37.9% males, aged 18 years and older. Overall age for participants was 43.2 ± 15.3 years.

Serological testing showed a high burden of hepatitis B and D (Table S1–S3). Anti-HBc was positive in 39.6% of cases, indicating past or current HBV infection. HBsAg was found in 4.7% (95% *CI:* 4.31–5.11%), with 68.1% of these showing active HBV DNA (Table S1-S2). HDV superinfection was present in 12.7% of HBsAg-positive individuals, when there were three HDV RNA positive cases among HBV DNA negative ones (Table S3).

**Fig. 1:**
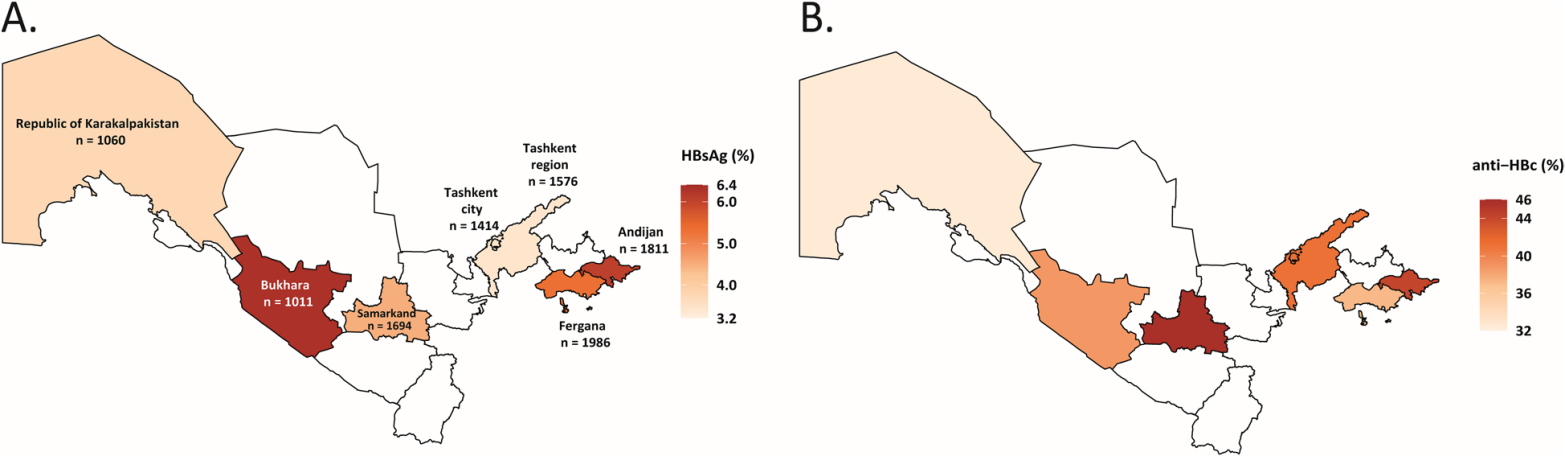
Regional choropleth maps of hepatitis B virus serological markers in Uzbekistan: **A** HBsAg positivity and **B** anti-HBc positivity. Shading indicates the observed prevalence in each study region, with darker colors representing higher values; the number of participants sampled in each region is shown as letter “n”. Regions not included in the study are displayed in white. Source: GeoJSON boundary from “github.com”, accessed on 09.06.2026. *HBsAg* Hepatitis B surface antigen; *anti-HBc* Hepatitis B core antigen

Geographic variation in hepatitis markers was significant (Fig. 1, Table S1–S3, Figure S1). Anti-HBc prevalence ranged from 32.5% to 45.9%, with Samarkand, Andijan, the Tashkent region, and Fergana showing higher odds than Tashkent city.

HBsAg prevalence demonstrated the most pronounced geographic clustering, ranging from 3.2% in Tashkent city to 6.3% in Bukhara, a 1.9-fold difference. Southern and eastern regions showed highest burden: Andijan (6.1%), Fergana (5.3%), and Bukhara (6.3%), while Tashkent region (3.4%) and Karakalpakistan (3.8%) demonstrated rates comparable to the capital. In multivariate analysis, only Bukhara remained significantly elevated (*aOR* 1.83, 95% *CI:* 1.21–2.77; *P* < 0.01).

HBV DNA prevalence among HBsAg-positive individuals ranged from 41.3% in Tashkent city to 77.8% in peripheral regions, indicating regional differences in disease progression (Table S2).

**Fig. 2:**
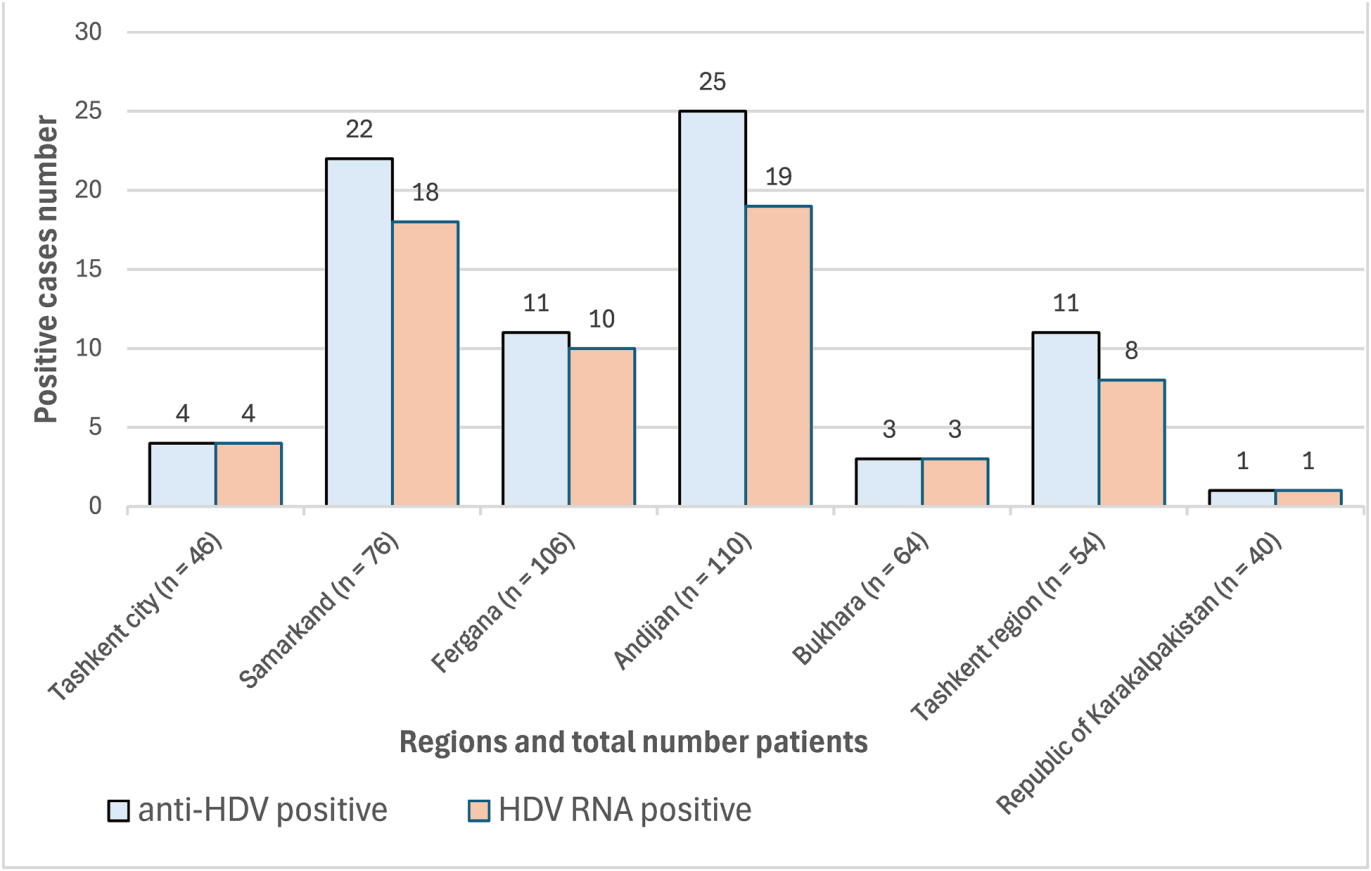
Regional distribution of anti-HDV-positive and HDV RNA-positive cases in Uzbekistan among HBV DNA-positive individuals; numbers in parentheses next to each region name on the x-axis denote the regional count of HBV DNA-positive cases. Light blue bars represent serological evidence of HDV exposure (anti-HDV positive); orange bars represent active viremic infection (HDV RNA positive). *HBV DNA* Hepatitis B virus deoxyribonucleic acid; *HDV RNA* Hepatitis D virus ribonucleic acid

HDV RNA prevalence among HBV DNA-positive individuals was highest in Samarkand (23.7%), well above the rate in Karakalpakistan (2.5%). Andijan had intermediate rates, while Bukhara had low HDV superinfection despite high HBV DNA prevalence (Fig. 2). Multivariate analysis confirmed Samarkand's elevated risk (*aOR* 3.34, 95% *CI:* 1.01–10.10; *P* = 0.05).

### Risk factors and associations with anti-HBc positivity

**Fig. 3:**
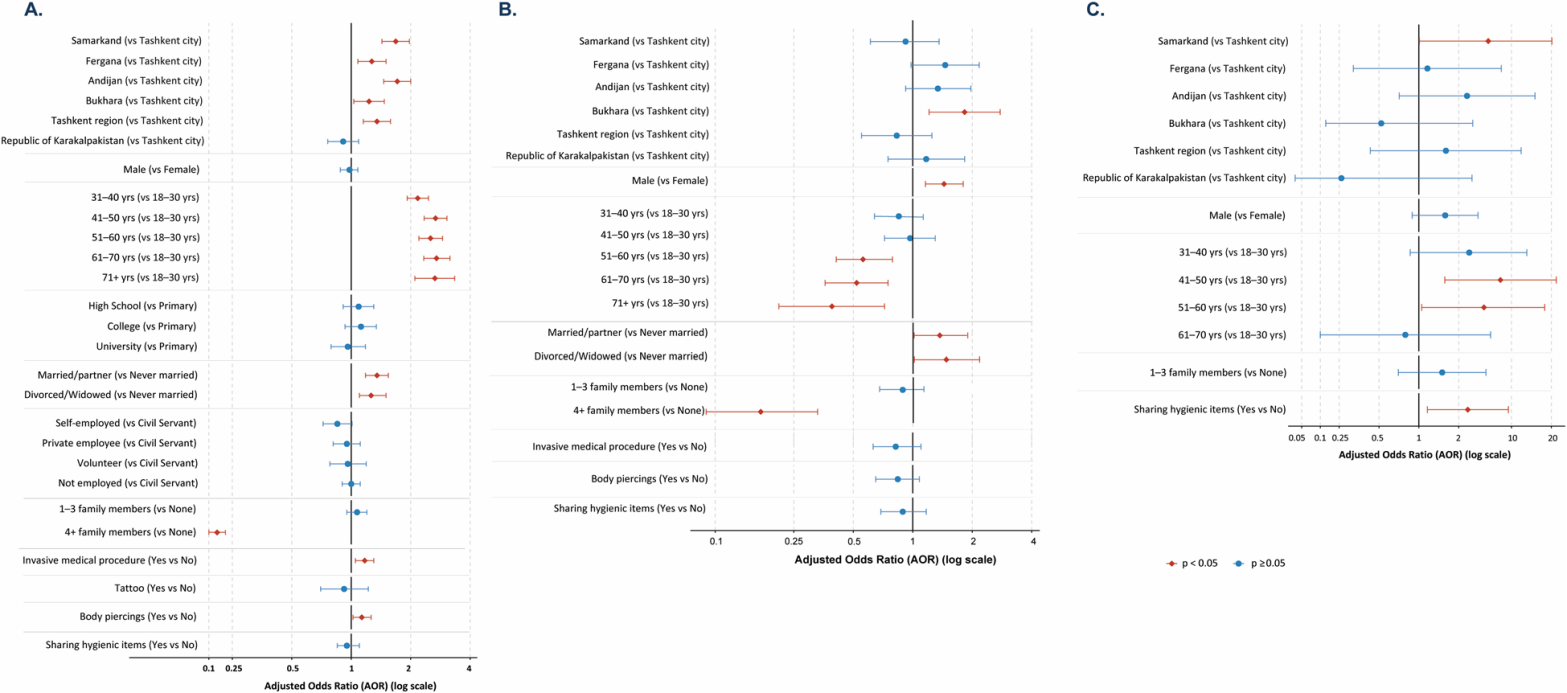
Forest plots of adjusted odds ratios for factors associated with anti-HBc positivity (**A**), HBsAg positivity (**B**), and HDV RNA positivity (**C**) in adults from seven regions of Uzbekistan. Points represent adjusted odds ratios, and horizontal bars indicate 95% confidence intervals on a logarithmic scale. Red indicates P < 0.05 and blue indicates P ≥ 0.05. Reference categories were Tashkent city, female sex, age 18–30 years, primary education, never married, civil servant, no family members, and absence of the exposure of interest. HDV RNA analyses were restricted to HBsAg-positive participants. For detailed information please see supplementary Table 4.* HBsAg* Hepatitis B surface antigen; *anti-HBc* Hepatitis B core antigen; *HDV RNA* Hepatitis D virus ribonucleic acid

Mean age of anti-HBc positivity group was 45.8 ± 14.3 years. Age was the strongest predictor of anti-HBc positivity. All older age groups had significantly higher odds than ages 18–30, reflecting cumulative exposure and the impact of vaccination introduced in later cohorts (Fig. 3A).

Sex was not significantly associated with anti-HBc prevalence, indicating similar exposure for males and females. Marital status was linked to higher anti-HBc risk, suggesting household or partner transmission. Household size demonstrated an unexpected protective effect. Participants with 4 or more family members had 86% lower odds of anti-HBc (a*OR* 0.14, 95% *CI*: 0.10–0.20; *P* < 0.01), whereas those with 1–3 family members showed no significant difference compared with those without family members.

A history of invasive medical procedures was linked to 17% increased odds of anti-HBc, indicating ongoing healthcare-associated transmission risk. Body piercings were modestly but significantly associated with 13% increased odds of anti-HBc (a*OR* 1.13, 95% *CI*: 1.02–1.26; *P* = 0.02). Univariate analysis showed a similar association (*OR *1.14, 95% *CI*: 1.05–1.25; *P* < 0.01) (Table S3).

Tattoos were not significantly associated with anti-HBc after adjustment. Sharing hygienic items was not significantly associated with anti-HBc positivity (Fig. 3A, Table S4).

Neither education level nor occupational status demonstrated significant associations with anti-HBc positivity in multivariate analysis (Table S4). Education effects were not significant across categories (high school: a*OR* 1.09, *P* = 0.37; college: a*OR* 1.12, *P* = 0.24; university: *aOR* 0.96, *P* = 0.72), and occupational categories showed no consistent associations with anti-HBc (Fig. 3A, Table S3).

### Risk factors and associations with HBsAg positivity

Mean age for HBsAg positive group was 42.8 ± 13.9 years. Unlike anti-HBc, HBsAg positivity decreased with age. Older adults had significantly lower odds, suggesting possible viral clearance, survival bias, or cohort effects (Table S4).

Females had 31% lower odds of HBsAg positivity than males, suggesting possible sex-based differences in disease clearance. Married or partnered individuals had higher odds of HBsAg positivity, as did divorced or widowed participants, suggesting household or partner transmission. Participants with 4 or more family members had 83% lower odds of HBsAg; households with fewer members showed no significant difference (Table S4).

The history of invasive medical procedures showed no significant association with HBsAg positivity (a*OR* 0.82, 95% *CI*: 0.63–1.10; *P* = 0.14, multivariate;* OR *0.8, 95% *CI*: 0.62–1.02; *P* = 0.07, univariate), suggesting that procedures increase exposure without necessarily establishing chronic infection (Table S3, Table S4).

Body piercings, tattoos, and sharing hygienic items were not significantly associated with HBsAg positivity (Table S4).

Education level and occupational status were excluded from HBsAg multivariate models because univariate *P*-values were > 0.25, indicating insufficient association strength to estimate independent effects (Fig. 3B, Table S3).

### Risk factors and associations with HDV RNA positivity

HDV RNA positivity was highest in middle-aged adults, especially ages 41–50, who accounted for 42.6% of cases. No cases were found in those aged 71 and older. HDV RNA positivity showed no significant sex difference. Household composition was not significantly associated with HDV RNA positivity. Samarkand had a notably higher HDV RNA positivity rate (23.7%) among HBV DNA-positive individuals; rates in other regions were not significant (Fig. 2).

Sharing of hygienic items demonstrated striking specificity for HDV RNA positivity, showing 134% increased odds (*aOR* 2.34, 95% *CI*: 1.16–4.74; *P* = 0.02). Remarkably, no significant association was observed with anti-HBc (*aOR* 0.95, 95% *CI*: 0.85–1.10; *P* = 0.39) or HBsAg (*aOR* 0.89, 95% *CI*: 0.69–1.17; *P* = 0.43). Univariate analysis confirmed this HDV-specific pattern (*OR* 2.33, 95% *CI:* 1.19–4.39; *P* = 0.01 for HDV). This striking differential association suggests that HDV transmission preferentially requires direct blood contact through microtrauma during personal hygiene activities, representing a distinct transmission pathway from HBsAg. Injection drug use was extremely rare (0.5% prevalence, *n* = 56 total), precluding meaningful multivariate analysis despite its known global importance as an HDV transmission route (Fig. 3C, Table S4).

Healthcare-associated transmission (invasive medical procedures), body piercings, tattoos, marital status, education, and occupational status showed no significant independent associations with HDV RNA positivity or were excluded because univariate *P*-values were > 0.25 (Fig. 3C, Table S3).

## Discussion

This population‑based study of 10,552 adults across seven regions of Uzbekistan reveals a substantial burden of hepatitis B and D infections, with important geographic heterogeneity and distinctive transmission patterns. The overall HBsAg prevalence of 4.7% places Uzbekistan in the intermediate endemicity category according to WHO classification and is broadly consistent with adult prevalence estimates from the nationwide screening cohort and earlier Uzbek surveys in general and intermediate‑risk populations [3, 20–22]. Anti‑HBc positivity in 39.6% of adults further underscores the large proportion of the population with past or current HBV exposure. When contextualised with data from neighboring Central Asian countries, which also report intermediate HBV endemicity, these findings support the regional comparability of our estimates and indicate that Uzbekistan remains part of a broader Central Asian hepatitis belt [23]. However, because the parent cohort was recruited through convenience sampling of healthcare attendees, individuals with low healthcare utilization and healthy asymptomatic adults may be under‑represented, and our seroprevalence estimates likely modestly overestimate the true prevalence in the general adult population; they are therefore best interpreted as representative of healthcare‑attending adults rather than a strict national census sample [8–10].

The 4.7% HBsAg prevalence observed in this adult healthcare‑attending population contrasts sharply with contemporary pediatric data from Uzbekistan, where HBsAg prevalence in schoolchildren is 0.2%, and with post‑2000 birth cohorts in the nationwide screening cohort, in which HBsAg prevalence has fallen to 0.8% compared with 3.2% in pre‑2000 cohorts [9, 20–22]. Together, these gradients indicate a clear generational transition: HBV infection has become rare among vaccinated younger cohorts, whereas chronic infection is increasingly concentrated among older, unvaccinated adults. This epidemiological shift implies that the main reservoir of chronic HBV—and the population in whom HDV superinfection and advanced liver disease will be concentrated over the coming decades—will be adults born before routine infant immunization, who should therefore be prioritized for targeted screening, linkage to care, and antiviral therapy [20–22].

The striking geographic clustering—with HBsAg prevalence ranging from 3.2% in Tashkent city to 6.3% in Bukhara—underscores persistent regional disparities in disease burden despite decades of national vaccination efforts [3, 14–16]. These patterns likely reflect a combination of historical transmission related to pre-vaccination healthcare practices and unsafe injections, regional heterogeneity in the timing and completeness of early vaccination program roll-out, and ongoing horizontal transmission through healthcare-associated and household exposures, as suggested by studies from Uzbekistan and comparable Central Asian and low- and middle-income settings [3, 9, 14–16, 20, 23–25]. Because detailed region-specific data on historic vaccination coverage and healthcare practices are limited, these explanations should be regarded as hypothesis-generating and warrant further investigation using more granular surveillance and molecular epidemiological approaches.

The finding that 68.1% of HBsAg-positive individuals had detectable HBV DNA indicates that most chronic carriers in this adult population have active viral replication. This highlights a substantial pool of infectious individuals who require clinical monitoring and, in many cases, antiviral therapy to prevent progression to cirrhosis and hepatocellular carcinoma. Most remarkably, this study identifies a potential HDV-specific transmission pathway through sharing of personal hygiene items that appears independent of HBsAg acquisition risk, suggesting distinct epidemiological dynamics for delta virus superinfection [24, 25].

The age‑related patterns observed for anti‑HBc and HBsAg prevalence provide compelling evidence for the impact of Uzbekistan’s newborn hepatitis B vaccination program, launched in 1998 and achieving full coverage by 2000 [20]. The higher anti‑HBc prevalence in older age groups reflects cumulative lifetime exposure in unvaccinated cohorts. In contrast, the decreasing HBsAg prevalence with advancing age likely reflects a combination of spontaneous viral clearance, survival bias, and, importantly, the protective effect of vaccination in younger cohorts. To aid interpretation of the age‑related patterns, the study age groups can be viewed in relation to Uzbekistan’s HBV vaccination timeline: participants aged 18–24 years during 2022–2024 were born largely in the vaccination era, those aged 25–30 years represent a transitional cohort, and those aged 31 years and older were predominantly born before routine infant immunization. This cohort mapping likely contributes to the lower HBV exposure markers observed in younger adults and the higher cumulative burden in older age groups [9, 26]. Studies from China and other high‑burden settings similarly document declining cohort effects, with individuals born after widespread HBV vaccination implementation showing substantially lower infection risk. The observed age-period-cohort patterns in our study are consistent with these trends, with younger adults likely benefiting from vaccination coverage that older cohorts did not receive. The intermediate prevalence observed in our adult population therefore contrasts sharply with the very low HBsAg prevalence among children and adolescents, underscoring the generational shift occurring as vaccinated cohorts mature and reinforcing the need to focus elimination strategies on older, unvaccinated adults who remain at highest risk and are most likely to harbor chronic infection [26, 27].

A second major finding is the marked geographic variation in both HBV and HDV burden across study regions. The pronounced geographic clustering of infections warrants careful interpretation. Bukhara region’s significantly elevated HBsAg prevalence (6.3%, *aOR* 1.83) relative to Tashkent city (3.2%) and the overall adult estimate (4.7%) indicates a localized excess of chronic HBV infection that is unlikely to be explained by random variation alone [3, 9]. Several non‑mutually exclusive mechanisms may underlie this pattern. First, historical transmission related to pre‑vaccination-era healthcare practices and unsafe injections—documented as major drivers of HBV transmission in Uzbekistan and other Central Asian and low‑ and middle‑income settings—may have generated a cohort of chronically infected adults who are disproportionately concentrated in Bukhara [3, 28]. Second, early implementation of the national HBV vaccination program likely showed regional heterogeneity, and delayed achievement of high infant coverage in some parts of Bukhara could have left specific birth cohorts incompletely protected, consistent with evidence of variable vaccine responsiveness and persistent inequities in immunization coverage across Central Asia and other LMICs [19, 45, 46]. Third, ongoing horizontal transmission through healthcare‑associated exposures and close household contact remains plausible, as studies from comparable settings show that suboptimal infection control, unsafe injections and intrafamilial clustering can sustain intermediate HBV endemicity even after vaccine introduction. In our data, the higher HBV DNA prevalence among HBsAg‑positive individuals in peripheral regions compared with Tashkent city (up to 77.8% vs 41.3%) is compatible with regional differences in disease stage, access to antiviral therapy, and possibly circulating genotype distribution [19, 24, 32]. Because detailed Bukhara‑specific information on historical vaccination coverage, healthcare practices and behavioral risk factors was not available, these region-level explanations should be regarded as hypothesis‑generating rather than definitive and warrant further investigation using finer‑grained surveillance and phylogenetic studies [3, 29].

The HDV RNA geographic pattern diverges notably from HBV, with Samarkand demonstrating strikingly high prevalence (23.7% of HBV DNA-positive individuals, *aOR* 3.34) while Bukhara shows low HDV superinfection despite elevated HBV burden. This geographic discordance between HBV and HDV suggests that delta virus transmission operates through additional or distinct pathways beyond those driving HBsAg acquisition. Central Asian HDV epidemiology remains understudied, though available evidence indicates heterogeneous prevalence across the region. The concentration of HDV in specific areas like Samarkand may reflect localized transmission networks, specific risk behaviors, or historical introduction patterns requiring targeted public health interventions [21, 22, 30].

The lower odds of HBsAg positivity among females merit separate consideration. The observation that females had 31% lower odds of HBsAg positivity compared to males (while showing no sex difference in anti-HBc prevalence) aligns with well-documented sexual dimorphism in chronic hepatitis B outcomes. Multiple mechanisms likely contribute to this pattern. Biologically, females demonstrate enhanced immune responses to HBV, mediated by higher TLR-7 expression on the X chromosome, stronger vaccine responses, and more robust T-cell activation. These immunological advantages facilitate more efficient viral clearance following acute infection, reducing progression to chronic carrier state [31–33].

Hormonal influences also play important roles, with estrogen enhancing antiviral immune responses while androgens may promote viral persistence through androgen response elements in the HBV genome. Studies consistently report that males with chronic HBV have higher viral loads, more rapid progression to cirrhosis and hepatocellular carcinoma, and lower rates of spontaneous HBsAg seroclearance compared to females. The similar anti-HBc prevalence between sexes in our study suggests comparable exposure rates, with sex differences emerging primarily during the transition from acute to chronic infection—a pattern consistent with differential viral clearance rather than differential exposure [34]. Gender-related factors beyond biology may also contribute, including healthcare-seeking behavior, occupational exposures, and cultural practices. However, the consistency of male predominance in chronic HBV across diverse global settings suggests biological mechanisms are primary.

Household-related findings require cautious interpretation because they include both expected and apparently paradoxical patterns. The striking inverse association between larger household size (≥ 4 family members) and both anti-HBc (*aOR* 0.14) and HBsAg (*aOR* 0.17) was an unexpected finding and should be interpreted cautiously, given that household contact is an established HBV transmission route. This inverse relationship contradicts conventional assumptions that larger households would facilitate transmission through increased contact frequency and shared personal items [25, 35–37].

Several possible explanations may account for this unexpected association. First, larger households in Uzbekistan may reflect younger family structures with children born after 1998, when universal HBV vaccination was implemented. If household size correlates with birth year of household members, the protective effect may represent a cohort effect, with larger families containing more vaccinated individuals who are protected from infection. Smaller households may disproportionately comprise older, unvaccinated adults at higher baseline risk [30, 38, 39]. This pattern may therefore be partly influenced by age structure, including a greater likelihood that larger households include vaccinated individuals.

Second, socioeconomic differences may also contribute to the observed association. Studies from other settings suggest larger family size can associate with better socioeconomic status, improved healthcare access, and higher vaccination coverage in some contexts, though the relationship varies by population. Alternatively, smaller households might represent isolated, unmarried, or divorced individuals with different risk profiles, including potential concentration of high-risk behaviors [39, 40]. Reverse causation, selection effects, or other unmeasured factors may also contribute. Individuals with chronic HBV infection may have reduced fertility or smaller family size due to chronic illness, creating a reverse causal pathway. This possibility is consistent with our finding that married individuals had higher odds of HBsAg positivity, suggesting household formation or partnership status may be a risk marker rather than household size per se [40–42].

This protective association with larger household size should nevertheless be interpreted cautiously, as it contrasts with substantial evidence that intrafamilial HBV transmission is common. Studies consistently identify household contacts as an important route of HBV transmission, including vertical transmission from mother to child and horizontal transmission among siblings or other close household contacts; serological evidence of past or current HBV infection has been reported in 38–45% of household contacts of chronic HBV carriers [43–45]. However, such studies generally assess transmission within households containing an identified index case and do not directly address the association between household size and infection risk at the population level[43–45]. Residual confounding by age structure, vaccination status, socioeconomic position and healthcare access cannot be excluded. Therefore, the inverse association observed in our study should be considered hypothesis-generating and requires confirmation in studies incorporating detailed household-level, longitudinal, and vaccination data.

The increased odds of both anti-HBc and HBsAg positivity among married or partnered individuals, and the elevated risk among divorced or widowed participants, strongly implicate household and sexual transmission pathways. Spousal transmission of HBV is well-documented, with studies reporting 12–33% HBsAg prevalence among spouses of chronic carriers—substantially higher than general population rates. Sexual transmission efficiency varies by direction, with unvaccinated women facing higher risk from infected male partners due to direct semen exposure, while men face lower (though still significant) risk from infected female partners except during menstruation when blood contact increases transmission probability [46, 47]. The association between marital status and infection likely reflects both sexual transmission and shared household exposures through personal items. Longitudinal studies demonstrate that transmission risk increases with marriage duration, as cumulative exposure time compounds infection probability. The elevated risk among divorced or widowed individuals may indicate that some marriages dissolved due to health complications from chronic HBV, or that these individuals experienced exposures during previous partnerships [47, 48]. These findings underscore the importance of pre-marital HBV screening and partner vaccination programs as prevention strategies. In countries implementing such programs, spousal transmission has been substantially reduced through identification of discordant couples and vaccination of susceptible partners [47–50].

Our findings also suggest that healthcare-related exposures remain relevant to HBV transmission, particularly as markers of past exposure. The association between history of invasive medical procedures and 17% increased anti-HBc odds (*aOR* 1.17, *P* < 0.01), coupled with the absence of significant association with HBsAg positivity, suggests that healthcare exposures contribute to HBV transmission but typically result in acute infections that are cleared rather than progressing to chronic carrier state. This pattern is biologically plausible, as healthcare-associated exposures in adults generally involve lower viral inocula compared to perinatal transmission, and adult immune systems clear HBV in over 90% of acute infections [1, 59].

Uzbekistan's healthcare system has undergone substantial improvements in infection control practices coinciding with the HBV vaccination program implementation around 2000. However, historical practices before widespread adoption of universal precautions, single-use syringes, and blood product screening likely contributed to transmission in older cohorts. Studies from Central Asia document that unsafe injection practices, inadequate sterilization, and reuse of medical equipment represented significant HBV transmission routes in the pre-2000 era [51, 52]. The persistent association with anti-HBc in our contemporary sample suggests ongoing, albeit reduced, transmission risk in healthcare settings. Healthcare-associated HBV transmission has been documented even in developed settings when infection control lapses occur. These findings support continued emphasis on universal precautions, proper sterilization protocols, and healthcare worker training to eliminate iatrogenic transmission [53].

Cosmetic exposures showed a mixed pattern, with body piercings but not tattoos associated with anti-HBc positivity. Body piercings showed a modest but significant association with anti-HBc positivity (*aOR* 1.13, *P* = 0.02) despite relatively low prevalence (2.9%) in our population. This finding aligns with meta-analytic evidence demonstrating body piercing as an independent risk factor for both HBV (pooled *OR* 1.80) and HCV (pooled *OR* 1.83) transmission. The biological mechanism involves percutaneous blood exposure through contaminated needles or equipment used sequentially on multiple clients without adequate sterilization [53–55]. The transmission risk varies substantially based on piercing setting and infection control practices. Professional piercing studios with proper sterilization protocols pose minimal risk, while informal settings without adequate equipment sterilization represent high-risk environments. The low prevalence of piercings in our Uzbek sample likely reflects cultural norms, but the significant association even at this low frequency suggests that individuals seeking piercings may cluster in settings with suboptimal infection control or represent populations with elevated risk profiles [54, 56, 57]. Interestingly, tattoos showed no significant association with either anti-HBc or HBsAg in multivariable analysis, despite similar biological transmission mechanisms. This may reflect different risk profiles between tattooing and piercing practices in Uzbekistan, recall bias, or insufficient statistical power given the sample size.

The HDV findings deserve separate attention because they differ substantially from the patterns observed for anti-HBc and HBsAg. One notable finding regarding HDV epidemiology is the highly specific association between sharing personal hygiene items and HDV RNA positivity (*aOR* 2.34, *P* = 0.02), with no comparable associations observed for anti-HBc (*aOR* 0.95, *P* = 0.39) or HBsAg (*aOR* 0.89, *P* = 0.43). This differential pattern suggests a potential HDV-specific transmission route in chronic HBV carriers that operate through distinct epidemiological pathways requiring more intimate blood contact than initial HBsAg acquisition. However, given the cross-sectional design of this study, temporality and causality cannot be established, and this finding should be interpreted as hypothesis-generating pending prospective or phylogenetic confirmation [58–60].

The biological plausibility of this potential HDV-specific route is supported by the requirement for direct blood-to-blood transmission through microtrauma during personal care activities. Items such as razors, toothbrushes, nail clippers, and other sharp personal grooming tools can harbor microscopic blood residues that remain infectious. HBV can survive on environmental surfaces for at least seven days, maintaining infectivity during this period. HDV, while requiring HBV co-infection for replication, may require higher infectious doses or more direct percutaneous inoculation than HBV alone, making shared personal items particularly efficient transmission vehicles among household members who already harbor chronic HBV [61, 62]. Previous molecular epidemiological studies support the plausibility of intrafamilial HDV transmission through close personal contact. Studies analyzing HDV genomic sequences demonstrate that family members infected with HDV carry nearly identical viral strains (average genetic divergence 2.0 ± 1.7% within families vs. 7.2 ± 2.2% among unrelated individuals, *P* < 0.01), providing molecular proof of household transmission through “inapparent permucosal or percutaneous passage of virus during close or intimate contact”. The familial clustering of HDV infections is significantly higher than for HBV alone, with 56% of HBsAg-positive family members of HDV-positive index cases also carrying HDV antibodies, compared to only 3% in control families (*P* < 0.01) [63, 64]**.**

The specificity of the hygienic item sharing association for HDV but not HBsAg may reflect several factors. First, the study population consists of adults, most with long-standing HBV infection acquired perinatally or in early childhood through vertical transmission or horizontal transmission from family members—routes that predominated before vaccination. Once individuals reach adulthood with established chronic HBV, their primary risk becomes HDV superinfection rather than new HBsAg acquisition. Second, HDV superinfection requires pre-existing HBV carrier state, creating a selected population already at risk through household contact with HBV-infected family members. The sharing of personal items within these households represents ongoing exposure opportunities specifically for HDV transmission [65, 66]. Third, the infectious dose and transmission efficiency may differ between HBV and HDV. While both viruses transmit through blood contact, HDV superinfection in chronic HBV carriers may require repeated or more intense exposures that daily sharing of contaminated personal items facilitates. This interpretation is supported by the concentration of HDV in specific geographic areas like Samarkand, suggesting localized transmission networks where cultural practices around personal item sharing may vary. The very low prevalence of injection drug use (0.5%) in our sample precluded meaningful analysis of this established high-risk HDV transmission route. In global epidemiology, people who inject drugs represent a major risk group for HDV superinfection due to direct blood exposure through shared injection equipment. However, the Uzbek general population appears to have different predominant exposure routes, with household-level transmission through personal item sharing playing a more prominent role [24, 67, 68].

While the analytical subsample was selected using stratified random sampling and is representative of the larger screening cohort, the parent cohort was recruited through convenience sampling at healthcare facilities, such that individuals with low healthcare utilization and healthy asymptomatic adults are likely underrepresented and the reported seroprevalence figures may modestly overestimate those in the true general adult population. Accordingly, the prevalence estimates reported here are likely more representative of healthcare-attending adults in Uzbekistan than of the general adult population and should be interpreted in that context when compared with community-based surveys. Second, the cross-sectional design prevents establishing temporal or causal relationships between risk factors and infection status. Third, self-reported behaviors are vulnerable to recall and social desirability bias, particularly for sensitive exposures such as injection drug use and sexual practices, and the questionnaire lacked detailed information on timing, frequency, and within-household infection dynamics. Fourth, absence of viral phylogenetic analyses limited confirmation of suspected transmission pathways suggested by epidemiological associations. Finally, residual confounding cannot be excluded despite multivariable adjustment; the inverse association with household size requires cautious interpretation due to complex links between family structure, age, and vaccination history, and the small number of HDV RNA-positive cases reduced power for subgroup analyses and may have obscured weaker risk factor associations [69]. No formal correction for multiple comparisons (e.g., Bonferroni or false discovery rate adjustment) was applied. Given the exploratory nature of risk factor analyses and the use of hypothesis-driven variable selection, this approach is consistent with common epidemiological practice; however, associations with borderline significance, particularly for HDV RNA outcomes with modest event numbers, should be interpreted with caution. Some observed associations may represent chance findings and warrant replication in independent datasets.

## Conclusions

This large, geographically diverse study demonstrates that Uzbekistan continues to face a substantial burden of chronic HBV infection and notable HDV superinfection in adults, with marked regional heterogeneity and distinct transmission patterns. Age-cohort effects confirm the major impact of the national HBV vaccination program, yet unvaccinated older adults remain a key reservoir requiring intensified screening and treatment. The identification of personal hygiene item sharing as a specific HDV transmission route, apparently independent of HBsAg acquisition, highlights an immediately actionable household-level prevention target. To advance toward viral hepatitis elimination goals, geographically targeted interventions in high-burden regions, family- and household-focused prevention, sustained high vaccination coverage, and ongoing surveillance are essential to maintain low prevalence in vaccinated cohorts and close remaining gaps across demographic groups.

## Supplementary Information

Supplementary Material 1

Supplementary Material 2

Supplementary Material 3

Supplementary Material 4

Supplementary Material 5

## Data Availability

The data set used and analyzed in the current study is available from the corresponding author on reasonable request.

## Abbreviations

a*OR*: Adjusted odds ratio
anti-HBc: Antibodies to hepatitis B core Antigen
anti-HDV: Antibodies to hepatitis Delta virus
*CI*: Confidence interval
DNA: Deoxyribonucleic acid
EIA: Enzyme immunoassay
HBsAg: Hepatitis B surface antigen
HBV: Hepatitis B virus
HBV DNA: Hepatitis B virus deoxyribonucleic acid
HBV + HDV: Hepatitis B and D co-infection
HCC: Hepatocellular carcinoma
HDV: Hepatitis D virus
HDV RNA: Hepatitis D virus ribonucleic acid
PCR: Polymerase chain reaction
TLR-7: Toll-Like receptor 7
WHO: World Health Organization
