# Supplementary material for: Hepatitis B and D burden in Uzbekistan: comprehensive analysis of seroprevalence and risk correlates from a countrywide study
Source: Infect Dis Poverty. 2026 Aug 17;15:88. doi: 10.1186/s40249-026-01487-w (PMC13479457; doi:10.1186/s40249-026-01487-w)
Supplement: Supplementary file 1 [file 40249_2026_1487_MOESM1_ESM.docx]

|  | anti-HBc positive | HBsAg postive |
| --- | --- | --- |
| Tashkent city (n=1414) | 33.6634 | 3.25318 |
| Samarkand (n=1694) | 45.9268 | 4.48642 |
| Fergana (n=1986) | 37.1601 | 5.33736 |
| Andijan (n=1811) | 44.1745 | 6.07399 |
| Bukhara (n=1011) | 38.7735 | 6.33037 |
| Tashkent region (n=1576) | 41.1802 | 3.4264 |
| Republic of Karakalpakistan (n=1060) | 32.4528 | 3.77358 |
| Total (n=10552) | 39.5849 | 4.70053 |

**Supplementary Figure 1.** *Prevalence of anti-HBc and HBsAg positivity by region in Uzbekistan.*

*Blue line indicates total HBV exposure (anti-HBc positive), orange line indicates active/chronic infection (HBsAg positive). Sample sizes per region shown in parentheses (n=10,532 total).*
