# Supplementary material for: Hepatitis B and D burden in Uzbekistan: comprehensive analysis of seroprevalence and risk correlates from a countrywide study
Source: Infect Dis Poverty. 2026 Aug 17;15:88. doi: 10.1186/s40249-026-01487-w (PMC13479457; doi:10.1186/s40249-026-01487-w)
Supplement: Supplementary file 2 [file 40249_2026_1487_MOESM2_ESM.docx]

| Supplementary table 1. HBsAg seroprevalence (%) with 95% confidence intervals by geographic region in Uzbekistan | | | |
| --- | --- | --- | --- |
| Region/City | **Total screened (n)** | **Number of positives** | **Prevalence (%, 95% CI)** |
| Tashkent city | 1,414 | 46 | 3.25% (2.42-4.36%) |
| Samarkand | 1,694 | 76 | 4.49% (3.61-5.58%) |
| Fergana | 1,986 | 106 | 5.34% (4.36-6.55%) |
| Andijan | 1,811 | 110 | 6.07% (5.02-7.32%) |
| Bukhara | 1,011 | 64 | 6.33% (4.99-8.01%) |
| Tashkent region | 1,576 | 54 | 3.43% (2.58-4.50%) |
| Republic of Karakalpakistan | 1,060 | 40 | 3.77% (2.63-5.29%) |
| Overall | **10,552** | **496** | **4.70% (4.31-5.11%)** |
| *This table presents the seroprevalence of hepatitis B surface antigen (HBsAg) across selected geographic regions of Uzbekistan, including the number screened, number of positive cases, and prevalence estimates with corresponding 95% confidence intervals*. | | | |
