# Supplementary material for: Hepatitis B and D burden in Uzbekistan: comprehensive analysis of seroprevalence and risk correlates from a countrywide study
Source: Infect Dis Poverty. 2026 Aug 17;15:88. doi: 10.1186/s40249-026-01487-w (PMC13479457; doi:10.1186/s40249-026-01487-w)
Supplement: Supplementary file 3 [file 40249_2026_1487_MOESM3_ESM.docx]

| Supplementary table 2.HBV DNA positivity (%) with 95% confidence intervals among HBsAg-positive individuals by geographic region in Uzbekistan | | | |
| --- | --- | --- | --- |
| Region/City | **Number of positives (HBsAg)** | **Number of HBV DNA-positive** | **Prevalence (%, 95% CI)** |
| Tashkent city | 46 | 19 | 41.30% (27.45-56.70%) |
| Samarkand | 76 | 44 | 57.89% (44.08-70.68%) |
| Fergana | 106 | 82 | 77.36% (69.42-84.13%) |
| Andijan | 110 | 73 | 66.36% (56.55-75.30%) |
| Bukhara | 64 | 47 | 73.44% (60.73-83.61%) |
| Tashkent region | 54 | 42 | 77.78% (65.37-86.94%) |
| Republic of Karakalpakistan | 40 | 31 | 77.50% (62.55-87.49%) |
| Overall | **496** | **338** | **68.15% (63.92-72.09%)** |
| *This table presents the proportion of individuals with detectable HBV DNA among HBsAg-positive participants across selected geographic regions of Uzbekistan, including the number of HBsAg-positive individuals, the number with detectable HBV DNA, and prevalence estimates with corresponding 95% confidence intervals.* | | | |
