# Supplementary material for: Hepatitis B and D burden in Uzbekistan: comprehensive analysis of seroprevalence and risk correlates from a countrywide study
Source: Infect Dis Poverty. 2026 Aug 17;15:88. doi: 10.1186/s40249-026-01487-w (PMC13479457; doi:10.1186/s40249-026-01487-w)
Supplement: Supplementary file 4 [file 40249_2026_1487_MOESM4_ESM.docx]

| *Supplementary table 3. Univariate logistic regression analysis of risk factors and sociodemographic characteristics associated with serological markers of hepatitis B and RNA of hepatitis D infection in 10,552 adult participants* *by geographic region in Uzbekistan.* | | | | | | | | | | | | | | | |
| --- | --- | --- | --- | --- | --- | --- | --- | --- | --- | --- | --- | --- | --- | --- | --- |
|  | | | ***Total***  ***(N = 10552)*** | ***Anti-HBc positive (n = 4177)*** | | | | ***HBsAg positive (n = 496)*** | | | | ***HDV RNA positive (n = 63)**** | | | |
|  | | | **Frequency (%)** | **Frequency (%)** | OR | [95% CI] | p-value | **Frequency (%)** | OR | [95% CI] | p-value | **Frequency (%)** | OR | [95% CI] | p-value |
| Tashkent city | | | 1414 (13.40) | 476 (11.40) | 1 | [Ref.] | - | 46 (9.27) | 1 | [Ref.] | - | 4 (6.34) | 1 | [Ref.] | - |
| Samarkand | | | 1694 (16.05) | 778 (18.63) | 1.67 | [1.44-1.93] | <0.01 | 76 (15.32) | 1.14 | [0.78-1.66] | 0.48 | 18 (28.57) | 3.18 | [1.0-10.10] | 0.03 |
| Fergana | | | 1986 (18.82) | 738 (17.67) | 1.16 | [1.01-1.34] | 0.04 | 106 (21.37) | 1.67 | [1.16-2.39] | <0.01 | 10 (15.87) | 1.06 | [0.31-3.60] | 0.91 |
| Andijan | | | 1811 (17.16) | 800 (19.15) | 1.56 | [1.35-1.8] | <0.01 | 110 (22.17) | 1.61 | [1.13-2.31] | <0.01 | 19 (30.15) | 2.14 | [0.68-6.68] | 0.16 |
| Bukhara | | | 1011 (9.58) | 392 (9.38) | 1.25 | [1.055-1.47] | <0.01 | 64 (12.90) | 1.81 | [1.22-2.69] | <0.01 | 3 (4.76) | 0.50 | [0.10-2.37] | 0.38 |
| Tashkent region | | | 1576 (14.94) | 649 (15.54) | 1.37 | [1.19-1.6] | <0.01 | 54 (10.88) | 0.94 | [0.63-1.41] | 0.79 | 8 (12.69) | 1.78 | [0.49-6.35] | 0.36 |
| Republic of Karakalpakistan | | | 1060 (10.05) | 344 (8.24) | 0.95 | [0.8-1.12] | 0.52 | 40 (8.06) | 1.25 | [0.81-1.93] | 0.30 | 1 (1.58) | 0.26 | [0.02-2.45] | 0.19 |
| Sex | *Female* | | 6549 (62.06) | 1512 (36.2) | 1 | [Ref.] | - | 225 (45.3) | 1 | [Ref.] | - | 34 (54.0) | 1 | [Ref.] | - |
|  | *Male* | | 4003 (37.94) | 2665 (63.8) | 1.1 | [1.02-1.19] | 0.01 | 271 (54.7) | 1.38 | [1.15-1.67] | <0.01 | 29 (46.0) | 1.49 | [0.88-2.55] | 0.14 |
| Age groups | *18–30* | | 2522 (23.90) | 577 (13.8) | 1 | [Ref.] | - | 94 (18.9) | 1 | [Ref.] | - | 5 (8.2) | 1 | [Ref.] | - |
|  | *31–40* | | 2605 (24.69) | 1115 (26.7) | 2.25 | [1.99-2.53] | <0.01 | 146 (29.5) | 0.89 | [0.68-1.18] | 0.44 | 19 (29.5) | 2.03 | [0.77-5.36] | 0.13 |
|  | *41–50* | | 1992 (18.88) | 948 (22.7) | 2.7 | [2.38-3.07] | <0.01 | 132 (26.5) | 1.01 | [0.77-1.34] | 0.93 | 27 (42.6) | 3.49 | [1.37-8.9] | <0.01 |
|  | *51–60* | | 1760 (16.68) | 810 (19.4) | 2.55 | [2.24-2.91] | <0.01 | 70 (14.1) | 0.59 | [0.43-0.82] | <0.01 | 10 (16.4) | 2.92 | [1.04-8.19] | 0.04 |
|  | *61–70* | | 1238 (11.73) | 551 (13.2) | 2.69 | [2.32-3.1] | <0.01 | 46 (9.3) | 0.56 | [0.39-0.79] | <0.01 | 2 (3.3) | 1.01 | [0.24-4.24] | 0.99 |
|  | *71 and older* | | 435 (4.12) | 176 (4.2) | 2.09 | [1.69-2.59] | <0.01 | 8 (1.7) | 0.34 | [0.19-0.61] | <0.01 | 0 | 1.4e-6 | - | 0.32 |
| Education | *Primary school* | | 664 (6.29) | 254 (6.1) | 1 | [Ref.] | - | 33 (6.7) | 1 | [Ref.] | - | 3 (4.8) | 1 | [Ref.] | - |
|  | *High School* | | 4116 (39.01) | 1724 (41.3) | 1.18 | [1-1.39] | 0.06 | 187 (37.6) | 0.81 | [0.55-1.18] | 0.27 | 25 (39.7) | 1.55 | [0.44-5.47] | 0.47 |
|  | *College* | | 3994 (37.85) | 1549 (37.1) | 1.04 | [0.88-1.24] | 0.61 | 201 (40.6) | 0.91 | [0.63-1.33] | 0.63 | 26 (41.3) | 0.52 | [0.42-5.22] | 0.52 |
|  | *University* | | 1778 (16.85) | 651 (15.6) | 0.91 | [0.76-1.09] | 0.33 | 75 (15.1) | 0.74 | [0.48-1.31] | 0.16 | 9 (14.3) | 0.94 | [0.24-4.5] | 0.94 |
| Marital status | *Never married* | | 1702 (16.13) | 451 (10.8) | 1 | [Ref.] | - | 54 (10.9) | 1 | [Ref.] | - | 6 (9.5) | 1 | [Ref.] | - |
|  | *Married or living with partner* | | 7493 (71.01) | 3187 (76.3) | 1.98 | [1.77-2.23] | <0.01 | 380 (76.6) | 1.36 | [1.03-1.81] | 0.03 | 47 (74.6) | 1.13 | [0.49-3.08] | 0.78 |
|  | *Divorced/Widowed* | | 1357 (12.86) | 539 (12.9) | 1.81 | [1.56-2.11] | <0.01 | 62 (12.5) | 1.12 | [0.93-1.93] | 0.12 | 10 (15.9) | 1.54 | [0.53-4.82] | 0.43 |
| Occupation | *Civil Servant* | | 2445 (23.17) | 993 (23.8) | 1 | [Ref.] | - | 114 (23.0) | 1 | [Ref.] | - | 17 (27.0) | 1 | [Ref.] | - |
|  | *Self employed* | | 918 (8.7) | 313 (7.5) | 0.75 | [0.64-0.88] | <0.01 | 48 (9.7) | 1.13 | [0.79-1.61] | 0.48 | 7 (11.1) | 0.96 | [0.38-2.53] | 0.96 |
|  | *Private company employee* | | 1043 (9.88) | 404 (9.7) | 0.94 | [0.80-1.09] | 0.38 | 56 (11.3) | 1.15 | [0.81-1.61] | 0.42 | 6 (9.5) | 0.68 | [0.25-1.85] | 0.44 |
|  | *Volunteer* | | 528 (5) | 187 (4.5) | 0.82 | [0.67-0.996] | 0.04 | 29 (5.9) | 1.03 | [0.68-1.57] | 0.89 | 4 (6.3) | 0.91 | [0.28-2.95] | 0.88 |
|  | *Not employed* | | 5618 (53.24) | 2280 (54.6) | 1.00 | [0.91-1.107] | 0.91 | 249 (50.1) | 0.88 | [0.69-1.1] | 0.28 | 29 (46.0) | 0.75 | [0.39-1.44] | 0.39 |
| Family members # | *Do not have* | | 2308 (21.87) | 912 (21.8) | 1 | [Ref.] | - | 127 (25.6) | 1 | [Ref.] | - | 28 (43.9) | 1 | [Ref.] | - |
|  | *1-3 members* | | 7761 (73.75) | 1893 (45.3) | 1.117 | [1.02-1.23] | 0.02 | 357 (71.9) | 0.75 | [0.60-0.93] | <0.01 | 35 (57.1) | 1.74 | [0.9-3.6] | 0.09 |
|  | *4 and more* | | 483 (4.58) | 110 (2.6) | 0.14 | [0.097-0.19] | <0.01 | 12 (2.4) | 0.17 | [0.09-0.29] | <0.01 | 0 | 0.58 | [0.27-1.1] | 0.09 |
| Had invasive medical procedure | | *No* | 8547 (81.0) | 3274 (82.7) | 1 | [Ref.] | - | 422 (85.1) | 1 | [Ref.] | - | 55 (87.3) | 1 | [Ref.] | - |
|  |  | *Yes* | 2005 (19.0) | 679 (17.3) | 1.21 | [1.09-1.34] | <0.01 | 74 (14.9) | 0.8 | [0.62-1.02] | 0.07 | 8 (12.7) | 1.01 | [0.34-1.68] | 0.58 |
| Have tattoo | | *No* | 10281 (97.43) | 3856 (98.0) | 1 | [Ref.] | - | 479 (96.6) | 1 | [Ref.] | - | 61 (96.8) | 1 | [Ref.] | - |
|  |  | *Yes* | 271 (2.57) | 77 (2.0) | 0.69 | [0.53-0.89] | <0.01 | 17 (3.4) | 1.15 | [0.7-1.89] | 0.58 | 2 (3.2) | 0.91 | [0.14-3.33] | 0.90 |
| Have body piercings | | *No* | 7479 (70.88) | 2878 (68.9) | 1 | [Ref.] | - | 368 (74.1) | 1 | [Ref.] | - | 50 (79.4) | 1 | [Ref.] | - |
|  |  | *Yes* | 3073 (29.12) | 1299 (31.1) | 1.14 | [1.05-1.25] | <0.01 | 128 (25.9) | 0.83 | [0.68-1.03] | 0.09 | 13 (20.6) | 0.71 | [0.36-1.33] | 0.30 |
| Sharing hygienic items | | *No* | 8874 (84.1) | 3555 (85.1) | 1 | [Ref.] | - | 430 (86.7) | 1 | [Ref.] | - | 48 (76.2) | 1 | [Ref.] | - |
|  |  | *Yes* | 1678 (15.9) | 622 (14.9) | 0.89 | [0.80-0.99] | 0.04 | 66 (13.3) | 0.82 | [0.63-1.06] | 0.12 | 15 (23.8) | 2.33 | [1.19-4.39] | 0.01 |
| Injected drugs | | *No* | 10496 (99.47) | 4156 (99.5) | 1 | [Ref.] | - | 493 (99.4) | 1 | [Ref.] | - | 63 (100) | 1 | [Ref.] | - |
|  |  | *Yes* | 56 (0.53) | 21 (0.5) | 0.85 | [0.49-1.47] | 0.55 | 3 (0.6) | 0.93 | [0.28-3.06] | 0.70 | 0 | 1.7e-6 | - | 0.36 |
| *HDV RNA positive cases among HbsAg positive cases  AOR, adjusted odds ratio; CI, confidence interval; Anti-Hbc, antibodies to the Hepatitis B core antigen; HBsAg, Hepatitis B surface antigen; HDV-RNA, Hepatitis D virus ribonucleic acid;  *For “Do not have” (Family Members) refers to participants who reported no family members residing in the same household at the time of the survey.*  *For "Volunteer" (Occupation) encompasses unpaid community service roles, NGO workers, and informal caregiving activities.* | | | | | | | | | | | | | | | |
| *Univariate logistic regression analysis of risk factors and sociodemographic characteristics associated with anti-HBc positivity, HBsAg positivity, and HDV RNA positivity in 10,552 adult participants across seven geographic regions in Uzbekistan. Results are presented as odds ratios (OR) with 95% confidence intervals [95% CI] and p-values. Reference categories are indicated as [Ref.]. *HDV RNA testing was performed only among HBsAg-positive individuals (n=496), with 63 testing positive for HDV RNA.* | | | | | | | | | | | | | | | |
