# Supplementary material for: Hepatitis B and D burden in Uzbekistan: comprehensive analysis of seroprevalence and risk correlates from a countrywide study
Source: Infect Dis Poverty. 2026 Aug 17;15:88. doi: 10.1186/s40249-026-01487-w (PMC13479457; doi:10.1186/s40249-026-01487-w)
Supplement: Supplementary file 5 [file 40249_2026_1487_MOESM5_ESM.docx]

| *Supplementary Table 4. Multivariate logistic regression analysis of risk factors and sociodemographic characteristics associated with serological markers of hepatitis B and RNA of hepatitis D infection in 10,552 adult participants by geographic region in Uzbekistan.* | | | | | | | | | | | | | | | |
| --- | --- | --- | --- | --- | --- | --- | --- | --- | --- | --- | --- | --- | --- | --- | --- |
|  | | | ***Total  (N = 10552)*** | ***Anti-HBc positive (n = 4177)*** | | | | ***HBsAg positive (n = 496)*** | | | | ***HDV RNA positive (n = 63) **** | | | |
|  | | | ***Frequency (%)*** | ***Frequency (%)*** | ***AOR*** | ***[95% CI]*** | ***p-value*** | ***Frequency (%)*** | ***AOR*** | ***[95% CI]*** | ***p-value*** | ***Frequency (%)*** | ***AOR*** | ***[95% CI]*** | ***p-value*** |
| *Tashkent city* | | | *1414 (13.40)* | *476 (11.40)* | *1* | *[Ref.]* | *-* | *46 (9.27)* | *1* | *[Ref.]* | *-* | *4 (6.34)* | *1* | *[Ref.]* | *-* |
| *Samarkand* | | | *1694 (16.05)* | *778 (18.63)* | *1.68* | *[1.43-1.97]* | *<0.01* | *76 (15.32)* | *0.92* | *[0.61-1.36]* | *0.67* | *18 (28.57)* | *3.34* | *[1.01-10.10]* | *0.05* |
| *Fergana* | | | *1986 (18.82)* | *738 (17.67)* | *1.27* | *[1.08-1.50]* | *<0.01* | *106 (21.37)* | *1.46* | *[0.98-2.17]* | *0.06* | *10 (15.87)* | *1.16* | *[0.32-4.19]* | *0.83* |
| *Andijan* | | | *1811 (17.16)* | *800 (19.15)* | *1.71* | *[1.46-2.00]* | *<0.01* | *110 (22.17)* | *1.34* | *[0.92-1.97]* | *0.13* | *19 (30.15)* | *2.31* | *[0.71-7.54]* | *0.16* |
| *Bukhara* | | | *1011 (9.58)* | *392 (9.38)* | *1.23* | *[1.03-1.47]* | *0.02* | *64 (12.90)* | *1.83* | *[1.21-2.77]* | *<0.01* | *3 (4.76)* | *0.52* | *[0.11-2.55]* | *0.42* |
| *Tashkent region* | | | *1576 (14.94)* | *649 (15.54)* | *1.35* | *[1.15-1.58]* | *<0.01* | *54 (10.88)* | *0.83* | *[0.55-1.25]* | *0.37* | *8 (12.69)* | *1.60* | *[0.43-5.92]* | *0.48* |
| *Republic of Karakalpakistan* | | | *1060 (10.05)* | *344 (8.24)* | *0.91* | *[0.76-1.09]* | *0.31* | *40 (8.06)* | *1.17* | *[0.75-1.83]* | *0.48* | *1 (1.58)* | *0.26* | *[0.03-2.52]* | *0.25* |
| *Sex* | ***Female*** | | *6549 (62.06)* | *1512 (36.2)* | *1* | *[Ref.]* | *-* | *225 (45.3)* | *1* | *[Ref.]* | *-* | *34 (54.0)* | *1* | *[Ref.]* | *-* |
|  | ***Male*** | | *4003 (37.94)* | *2665 (63.8)* | *0.98* | *[0.88-1.08]* | *0.72* | *271 (54.7)* | *1.44* | *[1.16-1.8]* | *<0.01* | *29 (46.0)* | *1.58* | *[0.89-2.8]* | *0.12* |
| *Age groups* | ***18–30*** | | *2522 (23.90)* | *577 (13.8)* | *1* | *[Ref.]* | *-* | *94 (18.9)* | *1* | *[Ref.]* | *-* | *5 (8.2)* | *1* | *[Ref.]* | *-* |
|  | ***31–40*** | | *2605 (24.69)* | *1115 (26.7)* | *2.17* | *[1.92-2.46]* | *<0.01* | *146 (29.5)* | *0.85* | *[0.64-1.13]* | *0.27* | *19 (29.5)* | *2.4* | *[0.86-6.53]* | *0.09* |
|  | ***41–50*** | | *1992 (18.88)* | *948 (22.7)* | *2.67* | *[2.34-3.05]* | *<0.01* | *132 (26.5)* | *0.97* | *[0.72-1.3]* | *0.85* | *27 (42.6)* | *4.13* | *[1.57-10.9]* | *<0.01* |
|  | ***51–60*** | | *1760 (16.68)* | *810 (19.4)* | *2.52* | *[2.20-2.9]* | *<0.01* | *70 (14.1)* | *0.56* | *[0.41-0.79]* | *<0.01* | *10 (16.4)* | *3.1* | *[1.05-8.91]* | *0.04* |
|  | ***61–70*** | | *1238 (11.73)* | *551 (13.2)* | *2.7* | *[2.33-3.16]* | *<0.01* | *46 (9.3)* | *0.52* | *[0.36-0.75]* | *<0.01* | *2 (3.3)* | *0.79* | *[0.18-3.48]* | *0.75* |
|  | ***71 and older*** | | *435 (4.12)* | *176 (4.2)* | *2.65* | *[2.10-3.34]* | *<0.01* | *8 (1.7)* | *0.39* | *[0.21-0.72]* | *<0.01* | *0* | *1.2e-6* |  | *0.99* |
| *Education* | ***Primary school*** | | *664 (6.29)* | *254 (6.1)* | *1* | *[Ref.]* | *-* | *33 (6.7)* | *1* | *[Ref.]* | *-* |  |  |  |  |
|  | ***High School*** | | *4116 (39.01)* | *1724 (41.3)* | *1.09* | *[0.91-1.3]* | *0.37* | *187 (37.6)* | *0.85* | *[0.57-1.26]* | *0.43* |  |  |  |  |
|  | ***College*** | | *3994 (37.85)* | *1549 (37.1)* | *1.12* | *[0.93-1.34]* | *0.24* | *201 (40.6)* | *1.10* | *[0.71-1.58]* | *0.77* |  |  |  |  |
|  | ***University*** | | *1778 (16.85)* | *651 (15.6)* | *0.96* | *[0.79-1.18]* | *0.72* | *75 (15.1)* | *0.89* | *[0.57-1.39]* | *0.62* |  |  |  |  |
| *Marital status* | ***Never married*** | | *1702 (16.13)* | *451 (10.8)* | *1* | *[Ref.]* | *-* | *54 (10.9)* | *1* | *[Ref.]* | *-* |  |  |  |  |
|  | ***Married or living with partner*** | | *7493 (71.01)* | *3187 (76.3)* | *1.35* | *[1.18-1.54]* | *<0.01* | *380 (76.6)* | *1.36* | *[1.00-1.85]* | *0.04* |  |  |  |  |
|  | ***Divorced/Widowed*** | | *1357 (12.86)* | *539 (12.9)* | *1.26* | *[1.10-1.50]* | *<0.01* | *62 (12.5)* | *1.48* | *[1.00-2.18]* | *0.046* |  |  |  |  |
| *Occupation* | ***Civil Servant*** | | *2445 (23.17)* | *936 (23.8)* | *1* | *[Ref.]* | *-* |  |  |  |  |  |  |  |  |
|  | ***Self employed*** | | *918 (8.7)* | *294 (7.5)* | *0.85* | *[0.72-1.01]* | *0.07* |  |  |  |  |  |  |  |  |
|  | ***Private company employee*** | | *1043 (9.88)* | *381 (9.7)* | *0.95* | *[0.81-1.11]* | *0.54* |  |  |  |  |  |  |  |  |
|  | ***Volunteer*** | | *528 (5)* | *175 (4.5)* | *0.96* | *[0.78-1.19]* | *0.72* |  |  |  |  |  |  |  |  |
|  | ***Not employed*** | | *5618 (53.24)* | *2146 (54.6)* | *1.00* | *[0.90-1.11]* | *0.97* |  |  |  |  |  |  |  |  |
| *Family members #* | ***Do not have*** | | *2308 (21.87)* | *859 (21.8)* | *1* | *[Ref.]* | *-* | *128 (25.6)* | *1* | *[Ref.]* | *-* | *28 (43.9)* | *1* | *[Ref.]* | *-* |
|  | ***1-3 members*** | | *7761 (73.75)* | *1782 (45.3)* | *1.07* | *[0.95-1.20]* | *0.24* | *357 (71.9)* | *0.89* | *[0.68-1.14]* | *0.35* | *35 (57.1)* | *1.5* | *[0.70-3.21]* | *0.29* |
|  | ***4 and more*** | | *483 (4.58)* | *101 (2.6)* | *0.14* | *[0.10-0.20]* | *<0.01* | *12 (2.4)* | *0.17* | *[0.09-0.33]* | *<0.01* | *0* |  |  |  |
| *Had invasive medical procedure* | | ***No*** | *8547 (81.0)* | *3274 (82.7)* | *1* | *[Ref.]* | *-* | *422 (85.1)* | *1* | *[Ref.]* | *-* |  |  |  |  |
|  |  | ***Yes*** | *2005 (19.0)* | *679 (17.3)* | *1.17* | *[1.05-1.30]* | *<0.01* | *74 (14.9)* | *0.82* | *[0.63-1.10]* | *0.14* |  |  |  |  |
| *Have tattoo* | | ***No*** | *10281 (97.43)* | *3856 (98.0)* | *1* | *[Ref.]* | *-* |  |  |  |  |  |  |  |  |
|  |  | ***Yes*** | *271 (2.57)* | *77 (2.0)* | *0.92* | *[0.70-1.22]* | *0.58* |  |  |  |  |  |  |  |  |
| *Have body piercings* | | ***No*** | *7479 (70.88)* | *2711 (68.9)* | *1* | *[Ref.]* | *-* | *368 (74.1)* | *1* | *[Ref.]* | *-* |  |  |  |  |
|  |  | ***Yes*** | *3073 (29.12)* | *1222 (31.1)* | *1.13* | *[1.02-1.26]* | *0.02* | *128 (25.9)* | *0.84* | *[0.65-1.08]* | *0.17* |  |  |  |  |
| *Sharing hygienic items* | | ***No*** | *8874 (84.1)* | *3555 (85.1)* | *1* | *[Ref.]* | *-* | *430 (86.7)* | *1* | *[Ref.]* | *-* | *48 (76.2)* | *1* | *[Ref.]* | *-* |
|  |  | ***Yes*** | *1678 (15.9)* | *622 (14.9)* | *0.95* | *[0.85-1.10]* | *0.39* | *66 (13.3)* | *0.89* | *[0.69-1.17]* | *0.43* | *15 (23.8)* | *2.34* | *[1.16-4.74]* | *0.02* |
| **HDV RNA positive cases among HbsAg positive cases The factors in grey cells of the table have p-value > 0.25, because of it there were not included in multivariable analysis AOR, adjusted odds ratio; CI, confidence interval; Anti-Hbc, antibodies to the Hepatitis B core antigen; HBsAg, Hepatitis B surface antigen; HDV-RNA, Hepatitis D virus ribonucleic acid;*  *For “Do not have” (Family Members) refers to participants who reported no family members residing in the same household at the time of the survey.*  *For "Volunteer" (Occupation) encompasses unpaid community service roles, NGO workers, and informal caregiving activities.*  *This table presents the multivariable logistic regression analysis of geographic, sociodemographic, and behavioral factors associated with anti-HBc seropositivity, HBsAg positivity, and HDV RNA positivity among 10,552 adults in Uzbekistan, reported as adjusted odds ratios with 95% confidence intervals and p-values; HDV RNA positivity was evaluated among HBsAg-positive participants only.* | | | | | | | | | | | | | | | |
